# Clinical efficacy and safety of multidrug therapy including thrice weekly intravenous amikacin administration for *Mycobacterium abscessus* pulmonary disease in outpatient settings: a case series

**DOI:** 10.1186/s12879-016-1689-6

**Published:** 2016-08-09

**Authors:** Ho Namkoong, Kozo Morimoto, Tomoyasu Nishimura, Hiromu Tanaka, Hiroaki Sugiura, Yoshitake Yamada, Atsuko Kurosaki, Takanori Asakura, Shoji Suzuki, Hiroshi Fujiwara, Kazuma Yagi, Makoto Ishii, Sadatomo Tasaka, Tomoko Betsuyaku, Yoshihiko Hoshino, Atsuyuki Kurashima, Naoki Hasegawa

**Affiliations:** 1 Division of Pulmonary Medicine, Department of Medicine, Keio University School of Medicine, Tokyo, Japan; 2 Japan Society for the Promotion of Science, Tokyo, Japan; 3 Department of Pulmonary Medicine, Eiju General Hospital, Tokyo, Japan; 4 Respiratory Disease Center, Fukujuji Hospital, Japan Anti-Tuberculosis Association, Tokyo, Japan; 5 Keio University Health Center, Tokyo, Japan; 6 Department of Diagnostic Radiology, Keio University School of Medicine, Tokyo, Japan; 7 Department of Diagnostic Radiology, Fukujuji Hospital, Japan Anti-Tuberculosis Association, Tokyo, Japan; 8 Center for Infectious Diseases and Infection Control, Keio University School of Medicine, 35 Shinanomachi, Shinjuku-ku Tokyo, 160-8582 Japan; 9 Leprosy Research Center, National Institute of Infectious Diseases, Tokyo, Japan

**Keywords:** *Mycobacterium abscessus*, Intravenous therapy, Amikacin, Rapid-growing mycobacterium, Outpatient treatment

## Abstract

**Background:**

*Mycobacterium abscessus* (*M. abscessus*) pulmonary disease is a refractory chronic infectious disease. Options for treating *M. abscessus* pulmonary disease are limited, especially in outpatient settings. Among parenteral antibiotics against *M. abscessus*, intravenous amikacin (AMK) is expected to be an effective outpatient antimicrobial therapy. This study evaluated the clinical efficacy and safety of intravenous AMK therapy in outpatients with *M. abscessus* pulmonary disease.

**Methods:**

This retrospective chart review of cases of *M. abscessus* pulmonary disease evaluated patient background data, AMK dosage and duration, sputum conversion, clinical symptoms radiological findings, and adverse events. *M. massiliense* was excluded on the basis of multiplex PCR assay.

**Results:**

Thirteen patients (2 men and 11 women) with *M. abscessus* pulmonary disease were enrolled at 2 hospitals. The median age at the initiation of intravenous AMK treatment was 65 years (range: 50–86 years). Patients received a median AMK dose of 12.5 mg/kg (range: 8.3–16.2 mg/kg) for a median duration of 4 months (range: 3–9 months). The addition of intravenous AMK led to sputum conversion in 10 of 13 patients, and 8 patients continued to have negative sputum status 1 year after treatment. Approximately half of the patients showed improvement on chest high-resolution computed tomography. There were no severe adverse events such as ototoxicity, vestibular toxicity, and renal toxicity.

**Conclusions:**

Thrice weekly intravenous AMK administration in outpatient settings is effective and safe for patients with *M. abscessus* pulmonary disease.

**Electronic supplementary material:**

The online version of this article (doi:10.1186/s12879-016-1689-6) contains supplementary material, which is available to authorized users.

## Background

The number of patients infected with *Mycobacterium abscessus* (*M. abscessus*), a rapidly growing mycobacterium, has been increasing recently [1, 2]. *M. abscessus* pulmonary disease is one of the most difficult bacterial infections to treat among nontuberculous mycobacterial (NTM) diseases because of its natural resistance to most available antibiotics. Although there are some treatment options for *M. abscessus* pulmonary disease, the 2007 ATS/IDSA statement about NTM states there are no drug regimens with proven or predictable efficacy and that the prognosis is poor [3]. Among available antibiotics, *M. abscessus* is typically sensitive to clarithromycin, amikacin (AMK), cefoxitin, and imipenem in vitro. Therefore, options for managing *M. abscessus* infection, a chronic incurable infectious disease, are limited especially in outpatient settings. Among parenteral agents, AMK is considered to be one of the most active agents against *M. abscessus* infection [4].

Accordingly, the 2007 ATS/IDSA statement about NTM mentions the clinical importance of long-term use of injectable AMK [3]. Regarding persistent AMK administration, previous studies on adverse events indicate daily systemic use can cause nephrotoxicity, ototoxicity, and vestibular toxicity [5]. For this reason, clinical studies on inhaled AMK aiming to overcome this clinical problem of adverse events have been initiated [6–8]. However, the practical use of intravenous AMK for patients with *M. abscessus* infection is an attractive alternative, especially in countries where inhaled AMK is not approved. Therefore, thrice weekly intravenous AMK adjusted according to a pharmacokinetics approach is an option for outpatient management for this disease. Furthermore, recent studies show *M. massiliense* exhibits better treatment response to clarithromycin [9, 10]. However, no case series have been reported on the clinical effects of this treatment approach in precisely diagnosed cases of *M. abscessus* infection, which excludes cases of *M. masilliense* infection.

Therefore, this retrospective case series of *M. abscessus* pulmonary disease at outpatient departments evaluated the clinical effects of intravenous AMK therapy.

## Methods

### Study population and data collection

The study population consisted of patients with *M. abscessus* pulmonary disease who received the combination therapy at Keio University Hospital (Tokyo, Japan) and Fukujuji Hospital (Tokyo, Japan) from January 2004 through December 2013. The diagnosis of *M. abscessus* pulmonary disease was based on the 2007 ATS/IDSA statement [3]. Among patients with *M. abscessus* pulmonary disease, those who received intravenous AMK therapy for more than 3 months were enrolled. We obtained informed consent from all these patients. This study was reviewed and approved by the research ethics committees of the Keio University School of Medicine and Fukujuji Hospital (2011-267-2, UMIN000007546).

### Amikacin administration

AMK was administered intravenously once per day thrice weekly for 30 min via the median cubital vein. When the patients visited clinics, peripheral intravenous lines were inserted and removed every time via the median vein. The targeted trough level of AMK is <1 μg/mL. Regarding the peak levels, the targeted levels is generally 56–64 μg/mL [11].

### Microbiological and renal function examination

Microbiological findings from lower respiratory tract specimens (i.e., sputum, bronchoalveolar lavage, and lung biopsy) were identified. The specimens were cultured on egg-based solid media (Kyokuto Pharmaceutical Industrial Co., Ltd. Tokyo, Japan) or mycobacteria growth indicator tubes (Becton, Dickinson and Co., Sparks, MD, USA). All isolates were identified as *Mycobacterium tuberculosis* or NTM by using the AccuProbe test (Gen-Probe Inc., San Diego, CA, USA). In addition, NTM species were identified by using the DNA–DNA hybridization test (Kyokuto Pharmaceutical Industrial Co., Ltd.). Multiplex PCR assay was used to distinguish *M. massiliense* from *M. abscessus* as described previously [12].

Sputum smears and cultures were evaluated 1 year after treatment completion. Sputum conversion was defined as 2 consecutive negative sputum cultures.

We also evaluated the influence of AMK administration on renal function by measuring serum creatinine concentration before treatment, 1 and 3 months after initiation of AMK, and at the end of treatment.

### Clinical symptoms

The presence/absence of fever, cough, fatigue, hemoptysis, weight loss, night sweats, dyspnea, and sputum production were evaluated before and after intravenous AMK administration by retrospective chart review and inquiry surveys to the patients following the previous study on inhaled AMK [6].

### Radiological examination

Chest high-resolution computed tomography (HRCT) images were evaluated by a radiologist and pulmonologist who were blinded to the clinical data. Discrepancies were resolved by consensus review. Radiological findings after AMK therapy were classified as “improved,” “unchanged,” or “worse.”

### Adverse events

The patients were monitored throughout the AMK therapy for peak and trough levels of AMK, nephrotoxicity, ototoxicity, and vestibular toxicity. Renal function was assessed monthly. We do not plan ahead for follow-up mandatory audiometry for all patients after AMK treatment as a protocol, but perform audiometry according to the physicians’ judgment, especially when the patient admitted has a hearing impairment or seek to be examined.

### Statistical analysis

Summary statistics for quantitative markers are presented as mean ± standard deviation as well as median (minimum, maximum). Differences were analyzed by paired *t*-tests and Fisher’s exact test using GraphPad Prism IV software (San Diego, CA).

## Results

**Fig. 1:**
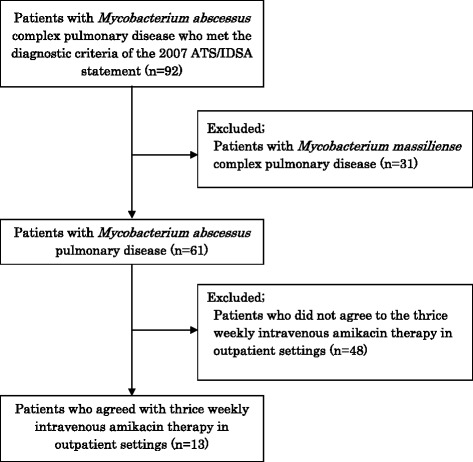
Flow diagram of patient enrollment

A total of 92 patients including 44 and 48 at Keio University Hospital and Fukujuji Hospital, respectively, who met the diagnostic criteria of the 2007 ATS/IDSA statement for *M. abscessus* complex pulmonary disease, including both *M. abscessus* and *M. massiliense*, were initially enrolled. We excluded 31 patients diagnosed with *M. massiliense* pulmonary disease on the basis of multiplex PCR assay. Among the 61 patients with *M. abscessus* pulmonary disease, 48 patients were not informed of or did not consent to thrice weekly intravenous AMK therapy in outpatient settings. Thus, 13 patients with *M. abscessus* pulmonary disease were successfully enrolled (Fig. 1). They did not receive AMK before the study period. The median duration of prior treatment was 16 months (range, 0–88 months).

**Fig. 2:**
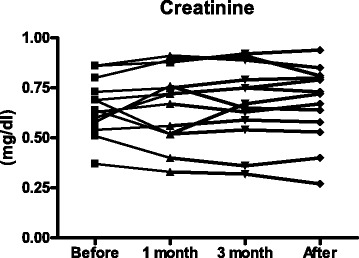
Serum creatinine concentrations before treatment, at 1 month after initiation of AMK, at 3 months after initiation of AMK, and at the end of treatment

All patients were negative for HIV. None of the patients were excluded during the observation period. Patient background data are shown in Table 1. There were 2 men and 11 women. The mean age at the initiation of intravenous AMK treatment was 63.6 ± 8.5 years (range, 50–86 years). Five patients exhibited coinfection of *Mycobacterium avium* complex (MAC) pulmonary disease. No patients underwent surgical resection of *M. abscessus* lesions. Among the study population, two patients had a smoking history (220 and 340 pack-years, respectively). None of the patients received long-term steroid therapy (>20 mg/day) or chest radiation. One patient with a breast cancer received chemotherapy in the past. Three had high soil exposure from farming and gardening. None of the patients were admitted for renal dysfunction before the initiation of AMK treatment (Fig. 2). All the 13 patients underwent audiometry before AMK treatment. No remarkable abnormal findings were obtained through the audiometric examinations.

**Table 1:** Patient background data

Backgrounds	(*n* = 13)
Age, mean ± SD (range), years	63.7 ± 8.5 (50–86)
Male/Female, no	2/11
Weight, mean ± SD (range), kg	44.7 ± 6.1 (35–56)
Smoking history, no (%)	2 (15.4)
Medical history, no (%)	
*Mycobacterium avium* complex pulmonary disease	5 (38.5)
Pulmonary aspergillosis	2 (15.4)
Hypertension	2 (15.4)
Dyslipidemia	2 (15.4)
Chronic obstructive pulmonary disease	1 (7.7)
Sarcoidosis	1 (7.7)
Chronic sinusitis	1 (7.7)
Gastric cancer	1 (7.7)
Breast cancer	1 (7.7)
Uterine myoma	1 (7.7)
Ovarian cyst	1 (7.7)

Although this is a retrospective case series, quite a number of patients with pulmonary *M. abscessus* disease did not receive AMK treatment. We compared patient background between the two groups in order to evaluate a potential bias (Additional file 1: Table S1). There were no significant differences between the two groups except body weight. Regarding renal function, creatinine in patients not receiving AMK was higher compared to that in patients receiving AMK, but not significantly.

During the observation period, all patients tolerated AMK therapy well and survived. Patients received intravenous AMK therapy for a median duration of 4 (range, 3–9) months. At the present, all the participants are not receiving AMK treatment.

Intravenous AMK therapy was originally initiated at a median dose of 15.2 (range, 13.8–16.2) mg · kg^−1^ · day^−1^ on hospitalization. The dosage was subsequently adjusted by measuring the trough drug concentrations. The trough level was considered the drug concentration measured just before the third administration, and the peak drug concentration was considered that 1 h after the third administration. In 8 patients, the trough level of AMK exceeded 1.0 μg/mL after the third administration, and the dose was decreased by a median 20.2 % (range, 18.2–21.4 %). The dose of AMK was also decreased in 2 of the 8 patients, because the measured level exceeded the target trough level even after the first dose reduction. After confirming the trough concentration was within a safe range, the predetermined dosage of AMK was subsequently administered at the outpatient department of the study hospitals (*n* = 7) or by home doctors (*n* = 6) in all cases. Patients eventually received a median dose of 12.5 (range, 8.3–16.2) mg/kg. The median peak level was 40.5 μg/mL (range, 18–72 μg/mL) after the third administration. As we mainly monitored trough levels of AMK as a safety measure in this study, we did not adjust the amount of intravenous AMK according to the peak levels.

All patients had positive mycobacterial smear results before starting intravenous AMK. Patients received an average of 6.1 months of treatment for mycobacterial infection before the initiation of intravenous AMK. Five patients who had coinfection of MAC received mycobacterial treatment without AMK. In addition, a few patients with *M. abscessus* pulmonary infection started treatment at first without intravenous treatment. At the end of AMK administration, 10 (77 %) of 13 patients exhibited negative sputum culture conversion. In 8 (62 %) of 13 patients, sputum culture remained negative for more than 1 year since the end of AMK administration. Two patients had subsequent positive sputum cultures at 4 and 8 months, respectively, after an initial negative culture.

The results of the drug susceptibility testing are shown in Additional file 2: Table S2. However, unfortunately, at present, in Japan, 7H9, not cation-adjusted Mueller-Hinton broth, which is recommended by the Clinical and Laboratory Standards Institute (CLSI), has been used in commercial drug susceptibility testing of *M. abscessus*. In addition, the pH used differed from the recommended pH (7.4) [13]. Although this result differs from the accurate definition of CLSI, all the cases were susceptible to AMK in this assay.

All patients were on a multidrug regimen when intravenous AMK was added. The 5 patients with coexistence of MAC pulmonary disease and those suspected of MAC pulmonary disease received clarithromycin, rifampicin, and ethambutol before the initiation of intravenous AMK. clarithromycin, sitafloxacin, and faropenem as oral agents, and imipenem/cilastatin and meropenem as parenteral agents were concomitantly used when intravenous AMK was started (Table 2).

**Table 2:** Clinical characteristics of patients with *Mycobacterium abscessus* receiving intravenous amikacin therapy

Case	Age	Body Weight (Kg)	Dose of AMK (mg)/(mg/kg)	Duration of AMK therapy(months)	Prior Treatment	Regimen at Initiation of AMK	Sputum Conversion after AMK therapy	Sputum Conversion 1 year after AMK therapy	Radiological Findings after AMK therapy
#1	68	37	400/10.8	6	CAM,EB,RFP	CAM,EB,RFP,FAM	-	-	Unchanged
#2	54	49.	600/12.2	3	CAM,EB,RFP,LVFX	CAM,FAM,STFX	+	+	Improved
#3	59	56	700/12.5	4	CAM,EB,RFP	CAM,FAM,STFX	+	+	Improved
#4	66	48	400/15.0	4	CAM,EB,RFP	CAM,FAM,STFX	+	+	Improved
#5	61	46	400/8.3	4	CAM,EB,RFP,SM	CAM,EB,FAM,STFX	+	+	Improved
#6	86	49	600/12.2	8	CAM,IPM/CS	CAM,FAM,LVFX	+	-	Improved
#7	50	36	500/13.9	9	-	CAM,FAM,MINO	+	+	Worsened
#8	56	40	600/15.0	4	-	CAM,IPM/CS,MINO	+	+	Improved
#9	62	52	750/14.4	4	CAM,EB,RFP,LVFX,	CAM,IPM/CS,MINO	+	+	Worsened
#10	65	46	600/13.0	4	CAM,EB,RFP,SM,STFX,FAM	CAM,RFP,FAM	+	+	Worsened
#11	69	43	700/16.2	4	-	CAM,IPM/CS	-	-	Worsened
#12	67	35	400/11.4	9	CAM,EB,RFP,SM	CAM,RFP,FAM	-	-	Unchanged
#13	65	44	400/9.1	6	CAM,EB,RFP	CAM,FAM	+	-	Improved

*M* male, *F* female, *CAM* clarithromycin, *EB* ethambutol, *RFP* rifampicin, *LVFX* levofloxacin, *SM* streptomycin, *STFX* sitafloxacin, *FAM* faropenem, *IPM/CS* imipenem/cilostazol, *MINO* minocycline

Fewer patients reported cough, sputum, dyspnea, hemoptysis, fever, and fatigue at the end of intravenous AMK than at baseline (Table 3). None of the patients reported night sweats.

**Table 3:** Clinical symptoms before and after amikacin treatment

Clinical symptom	Before treatment (*n* = 13)	After treatment (*n* = 13)	*P* value*
Cough	12 (92.3 %)	9 (69.2 %)	0.027
Sputum	11 (84.6 %)	7 (53.8 %)	0.182
Dyspnea	5 (38.5 %)	2 (15.4 %)	0.114
Hemoptysis	3 (23.1 %)	0 (0.0 %)	0.004
Fever	2 (15.4 %)	1 (7.7 %)	0.009
Fatigue	2 (15.4 %)	1 (7.7 %)	0.009
Night sweat	0 (0.0 %)	0 (0.0 %)	-

Data are presented as *n* (%) *:McNemar’s test

Seven of 13 patients exhibited improved chest HRCT findings after intravenous AMK treatment. At baseline, chest HRCT showed bronchiectasis and nodules, infiltration shadow, and cavities in 12, 8, and 4 patients, respectively. While findings of nodules and infiltration improved in 7 patients, bronchiectasis and cavities only improved in 1 patient after AMK treatment. Among the five patients with coinfection of MAC, three showed improvements (cases 3, 4, and 13 in Table 2), one showed deterioration (case 9 in Table 2), and one showed no change (case 1 in Table 2). The two patients (cases 1 and 9) were resistant to clarithromycin (minimum inhibitory concentration ≥32 μg/ml).

There were no severe adverse events such as ototoxicity and vestibular toxicity throughout the observation period. Of the 13 patients, 11 underwent audiometry after AMK treatment. None of the 11 patients had remarkable abnormal findings. The two patients who did not undergo follow-up audiometry did not complain of hearing disturbance after AMK treatment. Eight of 13 patients displayed the exceeded trough level of AMK (1 μg/ml) after the third administration as described before. Serum creatinine concentration was not influenced by AMK administration (Fig. 2).

## Discussion

This study evaluates the clinical effects of thrice weekly intravenous AMK on outpatients with *M. abscessus* pulmonary disease. Intravenous AMK therapy added onto existing regimens including clarithromycin was well tolerated by all patients. Furthermore, the addition of intravenous AMK led to negative sputum conversion in 10 (77 %) of 13 patients treated, which remained negative in 8 patients (62 %) 1 year after treatment. Moreover, approximately half of the patients showed improvements in chest HRCT findings. Therefore, the results suggest long-term intravenous AMK administered in outpatient settings is effective, safe, and clinically meaningful for *M. abscessus* pulmonary disease [11].

The strategy used to adjust AMK dose in this study should be highlighted. Peloquin et al. evaluated the toxicity of injectable aminoglycosides between daily and thrice weekly administration in several patients with NTM; in their trial using AMK 25 mg/kg thrice weekly, ototoxicity (37 %), nephrotoxicity (15 %), and vestibular toxicity (9 %) were highly prevalent [5]. Accordingly, the 2007 ATS/IDSA statement mentions that this dosage is impractical for intramuscular administration and therefore recommends a lower dosage [3]. In the present study, AMK administration was started at 15 mg/kg, and the dosage was subsequently adjusted by monitoring the trough concentration. Consequently, the median dose decreased to 12.5 (8.3–16.2) mg/kg. In fact, more than half of the patients underwent intravenous AMK dose reduction. This strategy is thought to be a major reason why no severe adverse events occurred.

Another strength of this study is the precise bacteriological diagnosis of *M. abscessus. M. massiliense* is a newly discovered *Mycobacterium* species. Unlike some previous studies involving clinical evaluations of *M. abscessus* [14–16], we evaluated strictly diagnosed *M. abscessus* pulmonary disease by excluding *M. massiliense* and *M. bolletii* using multiplex PCR assay.

The 2007 ATS/IDSA statement states negative sputum cultures for 1 year is unlikely for the treatment of *M. abscessus*. However, in the present study, negative sputum conversion lasted for 1 year in more than half of the patients; this is concordant with several other studies on *M. abscessus* pulmonary disease. For example, Lyu et al. report a case series of *M. abscessus* pulmonary disease treated with daily AMK in accordance with the 2007 ATS/IDSA statement [15]; the median duration of AMK administration was 230 (60–601) days, and 80 % of the patients achieved negative sputum conversion. Meanwhile, in the clinical study of Jarand et al., 71 % of enrolled patients received intravenous AMK for a median duration of 3 months, while 71 % of the patients exhibited negative sputum culture conversion [14]. Thus, these findings collectively indicate chemotherapy including intravenous AMK can achieve a higher percentage of sputum conversion.

In terms of susceptibility of *M. abscessus* in vitro, Park et al. reported that AMK was active against most isolates (99 %, 73/74), while imipenem (55 %, 36/66) and tobramycin (36 %, 27/74) were active against a moderate number of isolates [4]. Gayathri et al. also reported that of 148 rapidly growing mycobacterial isolates, 146 (98 %) were susceptible to AMK; and 138 (91 %), to gatifloxacin [17]. Based on these in vitro studies, AMK is thought to be the most reliable parental antibiotics against *M. abscessus*. The effectiveness of tigecycline and clofazimine has been reported recently and the synergic effects of these agents and AMK have been reported [18–20]. The efficacy of the combination therapy should be evaluated in clinical studies, and the optimal treatment should be determined.

Regarding chest HRCT findings, intravenous AMK treatment improved nodules and infiltrates in half of the patients in the present study. However, cavity and bronchiectasis tended to be irreversible lesions as reported in a radiological study of NTM [21]. Actually, as shown in Table 2, 5 out of 13 cases admitted worsened in chest HRCT findings in spite of AMK treatment. We reevaluated the clinical characteristics of the worsened cases. The worsened cases admitted the larger extent of bronchiectasis and larger cavity. Although we were not able to analyze statistically due to the small number, the extent of bronchiectasis and the size of cavity before the initiation of AMK treatment is thought to be one of the main factors which decides the succeed in posttreatment. Therefore, surgical removal should be strongly considered in operable situations, although no patients were operated on in the present study.

The duration of intravenous AMK therapy is controversial. Although long-term intravenous AMK administration is thought to be associated with better clinical outcomes, it could lead to a greater incidence of adverse events [21]. In addition, thrice weekly outpatient visits for many months would decrease patients’ quality of life. Accordingly, some recent studies of inhaled AMK for pulmonary NTM infections indicate inhaled AMK therapy leads to successful treatment without any evidence of systemic toxicity [6–8]. Therefore, inhaled AMK therapy is expected to be a new approach for pulmonary NTM infections. However, as mentioned above, inhaled AMK therapy is not approved in many countries besides the United States and some European countries. Nevertheless, the present results indicate our dose-adjusted intravenous AMK approach is very safe, practical, and feasible in countries where inhaled AMK therapy is not approved. Beyond the scope of this retrospective case series, successful intravenous AMK treatment requires home doctors to form good relationships with their patients. Accordingly, we asked the patients’ home doctors to prescribe the predetermined dosage of AMK in order to maintain treatment adherence and improve patient quality of life.

In this case series, we added AMK to other agents (faropenem, levofloxacin, etc.) simultaneously. When the patient has worsened clinical findings or is diagnosed with *M. abscessus* pulmonary disease, we considered modification of the chemotherapy or starting chemotherapy. We initiated AMK treatment during admission in order to adjust the AMK dose. As admission is suitable for the addition of other agents, AMK was added concomitantly to other agents just after the prior regimen.

We were unable to utilize cefoxitin, which is one of the standard antibiotics against *M. abscessus*, because of licensing issues in Japan. Accordingly, we expect the use of cefoxitin as an initial therapy will provide additional clinical benefits, because it is one of the few antibiotics to which *M. abscessus* is susceptible. Nevertheless, we cannot exclude the possibility of the clinical effects of the other concomitant agents. In particular, in addition to clarithromycin, we used combination chemotherapy with faropenem and sitafloxacin, which are not utilized outside Japan. Faropenem is an orally active beta-lactam antibiotic belonging to the penem group that is reported to exhibit considerable in vitro inhibitory activity against 56 strains of rapidly growing mycobacteria including *M. peregrinum*, *M. chelonae*, *M. fortuitum*, *and M. abscessus* [22]. Interestingly, a recent in vitro study reported that carbapenems, including faropenem, and rifampicin exhibit a synergic effect against *M. tuberculosis* and *M. abscessus* [23]. Accordingly, faropenem is widely used for *M. abscessus* pulmonary disease empirically in Japan. Sitafloxacin is a new a fluoroquinolone that has in vitro and in vivo activity against MAC and *Mycobacterium leprae* [24]; along with moxifloxacin, it is also reported to be active against *M. abscessus* [25]. In the near future, we plan to more comprehensively evaluate the clinical efficacy of these kinds of agents in conjunction with AMK.

In this retrospective study, we found five patients with coinfection of MAC. AMK has also been reported to be an active agent against MAC, including macrolide-resistant MAC. Recent studies have focused on the emergence of *M. abscessus* in MAC pulmonary disease [26]. Our study also highlights that AMK is an effective and safe agent for cases with coinfection of MAC and *M. abscessus*.

This study has some limitations that should be mentioned. First, clinical symptoms were evaluated by retrospective chart review and inquiry surveys to patients. Therefore, the improvement of clinical symptoms may have been overestimated, because we were unable to exclude recall bias. Another limitation is the evaluation of hearing ability as an adverse event related to AMK. We did not mandatory perform audiograms or vestibular examinations as a predecided protocol. Therefore, we may have overlooked minor decreases in hearing loss and vestibular toxicity. The other limitation is the chemotherapy regimen. We added AMK to other oral agents such as faropenem and sitafloxacin. Therefore, we could not evaluate the effectiveness of AMK alone against pulmonary *M. abscessus* diseases because of the presence of other potent agents. As the effectiveness of tigecycline and clofazimine has been recently reported [18–20], the efficacy of the combination therapy should be evaluated in clinical studies and the optimal treatment should be determined.

It is noteworthy that our study used AMK off-label, as the intravenous administration of AMK is not approved under the Japanese health insurance system at present. To overcome this ethical issue, this study was reviewed and approved by the respective Research Ethics Committees of Keio University School of Medicine and Fukujuji Hospital. We anticipate that antibiotics including AMK, which are potent against *M. abscessus*, will be approved as better treatment options in the near future.

## Conclusions

Thrice weekly intravenous AMK administration in combination with the regimen including clarithromycin in outpatient settings is effective and safe for patients with pulmonary *M. abscessus* disease.

## Abbreviations

AMK, amikacin; CLSI, Clinical and Laboratory Standards Institute; *M. abscessus*, *Mycobacterium abscessus*; MAC, *Mycobacterium avium* complex; NTM, nontuberculous mycobacteria

## Additional files

Additional file 1: Table S1. Comparison of patient characteristics between patients receiving AMK and patients not receiving AMK (DOCX 14 kb) Additional file 2: Table S2. Amikacin MICs of 13 patients with *Mycobacterium abscessus* (DOCX 13 kb)
