# Supplementary material for: Clinical efficacy and safety of multidrug therapy including thrice weekly intravenous amikacin administration for Mycobacterium abscessus pulmonary disease in outpatient settings: a case series
Source: BMC Infect Dis. 2016 Aug 9;16:396. doi: 10.1186/s12879-016-1689-6 (PMC4977760; doi:10.1186/s12879-016-1689-6)
Supplement: Additional file 1: Table S1. — Comparison of patient characteristics between patients receiving AMK and patients not receiving AMK (DOCX 14 kb) [file 12879_2016_1689_MOESM1_ESM.docx]

Additional file 1: Table S1

Comparison of patient characteristics between patients receiving AMK and patients not receiving AMK

| Characteristics | Patients receiving AMK (n=13) | Patients not receiving AMK (n=48) | P value |
| --- | --- | --- | --- |
| Age, years | 63.7 ± 8.5 | 66.5 ± 13.4 | 0.486^a^ |
| Male / Female, no (%) | 2 (15.4) / 11 (84.6) | 14 (29.2) / 34 (70.8) | 0.267^b^ |
| Weight, kg | 44.7 ± 6.1 | 53.5 ± 8.1 | 0.001^a^ |
| Smoking history, no (%) | 2 (15.4) | 10 (20.8) | 0.502 ^b^ |
| Cre, mg/dl | 0.65 ± 0.14 | 0.74 ± 0.27 | 0.156 ^a^ |

Data are presented as mean ± SD (standard deviation) unless otherwise indicated. AMK: amikacin a: Student’s t-test, b: Fisher's exact test
