# Supplementary material for: Clinical efficacy and safety of multidrug therapy including thrice weekly intravenous amikacin administration for Mycobacterium abscessus pulmonary disease in outpatient settings: a case series
Source: BMC Infect Dis. 2016 Aug 9;16:396. doi: 10.1186/s12879-016-1689-6 (PMC4977760; doi:10.1186/s12879-016-1689-6)
Supplement: Additional file 2: Table S2. — Amikacin MICs of 13 patients with Mycobacterium abscessus (DOCX 13 kb) [file 12879_2016_1689_MOESM2_ESM.docx]

Additional file 2: Table S2

Amikacin MICs of 13 patients with *Mycobacterium abscessus*

| MIC | n |
| --- | --- |
| 4 µg/mL | 2 |
| 8 µg/mL | 4 |
| 16 µg/mL | 7 |

MIC; minimum inhibitory concentration
